# Developing a support intervention for family members of people treated under the Mental Health Act

**DOI:** 10.1192/j.eurpsy.2024.564

**Published:** 2024-08-27

**Authors:** I. Wells, K. Wintsch, D. Giacco

**Affiliations:** ^1^University of Warwick, Coventry; ^2^Queen Mary University of London, London, United Kingdom

## Abstract

**Introduction:**

In England, a national Mental Health Act (MHA) review was carried out, providing recommendation for policy and practice changes. One of these recommendations was to provide support to family members (FMs) of patients who have been involuntarily hospitalised. In response to this review, the National Institute for Health and Care Research (NIHR) provided funding for research to address policy questions, for example what support may be most relevant to FMs during their patients’ treatment. The study presented here was funded as part of this initiative and has explored what support FMs wish to receive.

**Objectives:**

To explore FMs’ experiences and views around support they would have liked during their patients’ involuntary hospitalisation and wider stakeholder views on what a family support programme in England should involve. This information can be used to develop a support programme for FMs.

**Methods:**

One-to-one interviews were conducted online as part of two work packages. The first work package explored FMs’ experiences and views of support. The second work package explored views on what should be included in a family support programme in England from FMs, patients and professionals. Interviews took place at three sites across England. Audio recordings of the interviews were transcribed, and data were analysed using thematic analysis.

**Results:**

22 FMs were recruited to the first work package, where four key themes were identified: (1) heterogeneity in the current support for families; (2) information about mental health and mental health services; (3) continuous support; and (4) peer support and guidance. FMs reported receiving support from professionals, peers and relatives, but the extent of this support varied. FMs consistently reported wanting a named contact to provide information and personal continuity of support. 5 FMs, 4 patients and 10 professionals took part in the second work package, where four main themes were also identified: (1) development of a support programme; (2) delivery of a support programme; (3) factors limiting accessibility or engagement; and (4) benefits of a support programme. Information about the MHA and strategies to promote effective communication between FMs and professionals were identified as important to include in the programme and its delivery should include a combination of face-to-face, written and online methods. Potential benefits of the programme include improved FM knowledge and wellbeing.

**Conclusions:**

FMs of involuntarily hospitalised patients should receive information around the MHA and strategies to promote effective communication with professionals. FMs should also be allocated a named contact person to offer information and personal continuity of support through various methods, for example through online, face-to-face or written contact.

**Disclosure of Interest:**

None Declared

